# Ratiometric Monitoring of Biogenic Amines by a Simple Ammonia-Response Aiegen

**DOI:** 10.3390/foods11070932

**Published:** 2022-03-24

**Authors:** Xujing Guo, Xirui Chen, Rui Chen, Yujie Tu, Tianying Lu, Yuqian Guo, Liang Guo, Yonghua Xiong, Xiaolin Huang, Ben Zhong Tang

**Affiliations:** 1 State Key Laboratory of Food Science and Technology, School of Food Science and Technology, Nanchang University, Nanchang 330031, China; 18735430253@163.com (X.G.); cxirui219921@163.com (X.C.); tianyingluai@163.com (T.L.); yuqianguoncu@163.com (Y.G.); yhxiongchen@163.com (Y.X.); 2 Jiangxi-OAI Joint Research Institute, Nanchang University, Nanchang 330047, China; bioguo@163.com; 3 Key Laboratory of Clinical Laboratory Diagnostics (Ministry of Education), College of Laboratory Medicine, Chongqing Medical University, Chongqing 400016, China; 4 AIE Institute, Guangzhou Development District, Guangzhou 510530, China; tuyj@aietech.org.cn (Y.T.); tangbenz@cuhk.edu.cn (B.Z.T.); 5 Shenzhen Institute of Aggregate Science and Technology, School of Science and Engineering, The Chinese University of Hong Kong, Shenzhen 518172, China

**Keywords:** aggregation-induced emission, fluorescent sensor, paper chip, biogenic amines, food spoilage

## Abstract

Herein, we developed a paper-based smart sensing chip for the real-time, visual, and non-destructive monitoring of food freshness using a ratiometric aggregation-induced emission (AIE) luminogen (i.e., H^+^MQ, protonated 4-(triphenylamine)styryl)quinoxalin-2(1*H*)-one) as pH sensitive indicators. Upon exposure to amine vapors, the deprotonation of H^+^MQ occurs and triggers its color change from blue to yellow, with the fluorescence redshift from blue to amaranth. Consequently, we successfully achieved the sensitive detection of ammonia vapors by recording the bimodal color and fluorescence changes. Given the high sensitivity of H^+^MQ to ammonia vapor, a paper-based smart sensor chip was prepared by depositing H^+^MQ on the commercial qualitative filter paper through a physical deposition strategy. After being placed inside the sealed containers, the developed H^+^MQ-loaded paper chip was applied to the real-time monitoring of biogenic amine contents according to its color difference and ratio fluorescence change. The detection results were further compared with those obtained by the high-performance liquid chromatography method, which verified the feasibility of the designed paper chip for the food spoilage degree evaluation. Briefly, this work indicates that the designed H^+^MQ-loaded paper chip could be a promising approach for improving food freshness monitoring.

## 1. Introduction

Meat and seafood are widely loved by consumers as major sources of protein, minerals, and vitamins [1,2,3]. Due to the restrictions of production and breeding areas, they often need to be transported all over the world [4]. During the long-term transportation and storage, inappropriate conditions, such as incorrect temperature, may cause their corruption and deterioration [5,6], thus resulting in low quality products. Generally, the freshness of meat and seafood can be judged by sensory score [7], color difference value, texture index [8,9], total colony count [10], pH value [11], and biogenic amine content [12]. Among them, biogenic amines (BAs) are widely regarded as direct indicators of food deterioration, owing to their abnormal accumulation from the degradation of amino acids and the amination of organic compounds during food spoilage [13]. Therefore, to reduce the adverse effects of BAs on consumer health, the detection of the biogenic amine content in food products is of great significance to their freshness monitoring and quality evaluation.

Currently, the available methods for the detection of BAs mainly include high performance liquid chromatography (HPLC) [14], gas chromatography mass spectrometry [15,16], capillary electrophoresis [17], electrochemical systems [18,19], electronic nose [20,21,22], and electronic tongue [23]. However, their widespread use in biogenic amine monitoring is greatly limited by the requirement of expensive and sophisticated instruments, skilled technicians, time-consuming operation, and the destructive nature of the samples [24,25]. In recent years, fluorescence sensing technology has attracted more and more attention in biogenic amine monitoring and freshness evaluation due to its outstanding advantages, such as a high sensitivity, simple operation, fast response speed, low cost, real-time visualization, and non-destructive monitoring [26]. However, most of the fluorescence sensors reported so far rely on traditional organic fluorophore as an indicator, and their fluorescence signals significantly decrease in the solid or aggregated states owing to the aggregation-caused quenching (ACQ) effect [27], thus causing poor sensing performances, especially in solid-state sensor applications.

Unlike traditional ACQ fluorophores, fluorogens with aggregation-induced emission properties (AIEgens) exhibit enhanced emission in solid or aggregated states, making them suitable as high-performance solid-state fluorescent indicators [28,29,30,31,32]. Taking advantage of this property, scholars have developed various types of biogenic amine-response AIEgens and demonstrated their use in food freshness monitoring [33,34,35,36]. However, these reported AIEgens rely mainly on changes in absolute fluorescence intensity and perform poorly because the detection accuracy is susceptible to factors unrelated to the analyte, such as fluorogen concentration, instrument parameters, background scattering light and auto-fluorescence, and external environment variations (e.g., temperature and humidity) [37,38]. In this context, the emergence of ratiometric fluorescent system shows its great potential as an ideal optical sensor, because ratiometric measurement functions in self-calibrating signal correction and can enable more accurate detection [39,40,41,42].

Herein, we report on a paper-based ratiometric fluorescent sensing chip that uses a single AIEgen (i.e., H^+^MQ, protonated 4-(triphenylamine)styryl)quinoxalin-2(1*H*)-one) as a biogenic-amine-response indicator and demonstrate its great potential for real-time, visual, non-destructive monitoring of meat and seafood freshness. When exposed to amine vapor, the H^+^MQ molecule is prone to deprotonation and thus regulates its optical behaviors by altering the donor (D)−acceptor (A) interaction through the intramolecular charge transfer (ICT) effect. After the biogenic-amine reaction, the initial color of the prepared paper chip changed from blue to yellow, with its fluorescence red shift from blue to amaranth. The developed AIEgen-based paper chip can not only provide a fast and reversible bimodal colorimetric and fluorescence response to ammonia vapor, but can also realize real-time visualization of the ammonia volatilization process, indicating that the paper chip can be used for real-time, visual, non-destructive biogenic amine monitoring and freshness evaluation. By recording the color difference and ratio of fluorescence change, the relationship between ΔE and FI_570nm_/FI_410nm_ of paper chips and food freshness was built by simultaneously analyzing the biogenic amine index (BAI) in chicken, fish, and shrimp at different storage temperatures (25 °C, 4 °C, and −20 °C). The detection results were further compared with those obtained by the HPLC method. Collectively, this work provides a promising AIEgen-based ratiometric smart sensor chip for improving food freshness monitoring.

## 2. Materials and Methods

### 2.1. Materials

Qualitative filter paper, HCl, NaOH, NaHCO_3_, NaCl, sucrose, glycerinum, ammonia solution (25–28%), dimethyl sulfoxide, dimethylformamide, tetrahydrofuran, ethanol, methanol, chloroform, dichloromethane, acetonitrile, diethyl ether, acetone, *n*-butanol, *n*-hexane, propyl alcohol, acetic acid, ammonium acetate, sodium glutamate, and trichloroacetic acid were purchased from Sinopharm Chemical Reagent Co., Ltd. (Shanghai, China). Dansyl chloride, histamine hydrochloride, *β*-phenethylamine hydrochloride, tyramine hydrochloride, putrescine hydrochloride, cadaverine hydrochloride, tryptamine hydrochloride, spermine hydrochloride, spermidine hydrochloride, octopamine hydrochloride, and 1,7-diaminoheptane were bought from Aladdin Reagent Inc. (Shanghai, China). Fresh chicken, weeverfish, and shrimp were obtained from the local supermarket (Nanchang, China).

### 2.2. Preparation of H^+^MQ-Loaded Paper Chip

**Scheme 1:**
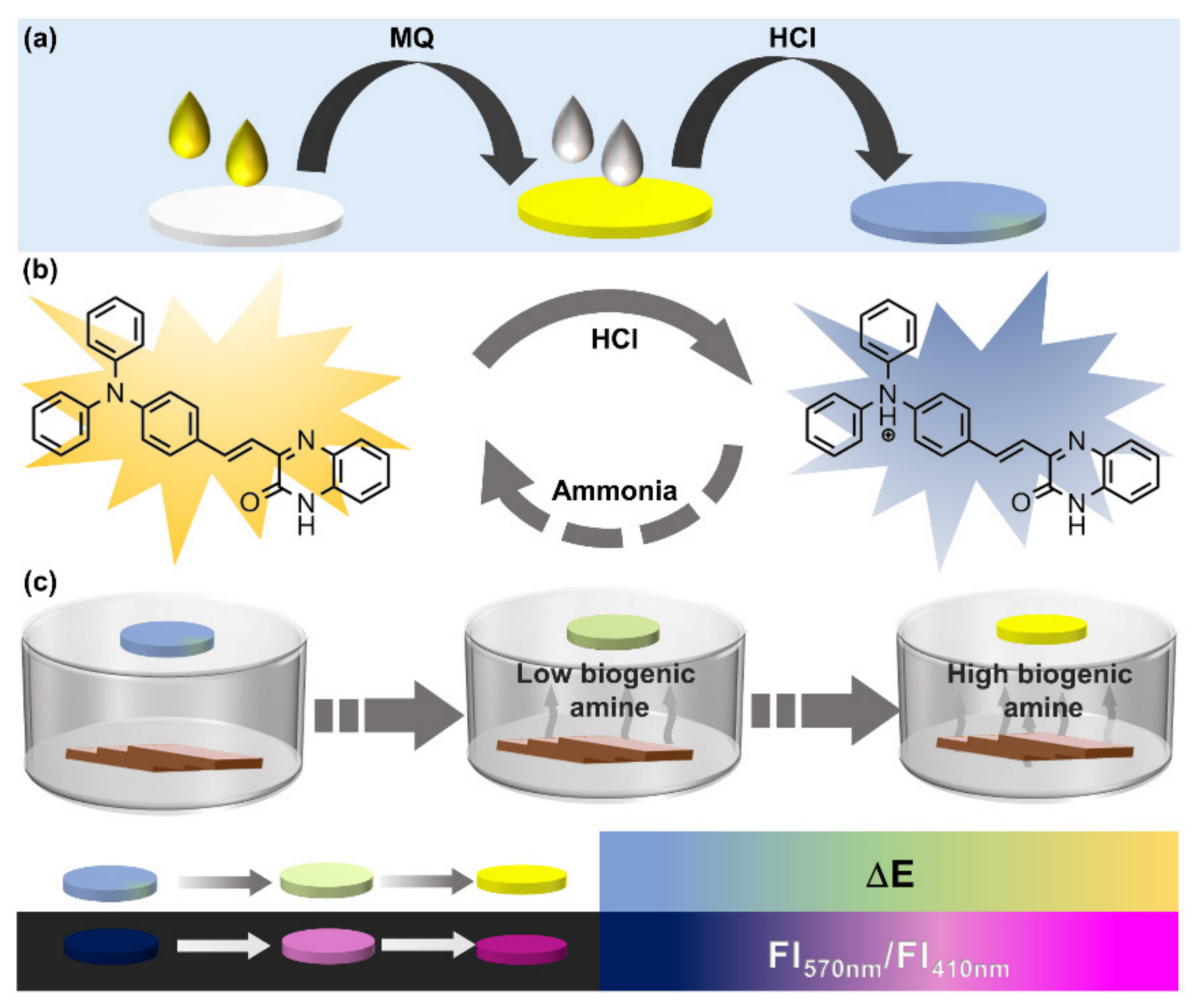
(**a**) Schematic illustration of the preparation process of a H^+^MQ-loaded paper chip. (**b**) The protonation and deprotonation process of MQ in the presence of HCl and ammonia. (**c**) Real-time and visual monitoring of BAs for food freshness evaluation using the proposed H^+^MQ -loaded paper chip.

Scheme 1a depicts the preparation of H^+^MQ-loaded paper chips. Firstly, MQ was dissolved in dichloromethane with a concentration of 0.5 mg/mL. Then, 3 μL of as-prepared MQ solution was dropped onto the qualitative filter paper. After drying the filter paper for 2 h at 25 °C, 5 μL of HCl solution (3 M) was added dropwise onto the filter paper. After being dried overnight at 25 °C, the H^+^MQ-loaded paper chip was cut into 1 × 1 cm pieces and stored at 4 °C until further use.

### 2.3. Ammonia Response of H^+^MQ-Loaded Paper Chip

The response behaviors of the H^+^MQ-loaded paper chip to ammonia were determined through treatment with different concentrations of ammonia. The color difference (ΔE) and FI_570nm_/FI_410nm_ of the H^+^MQ-loaded paper chip were recorded, where ΔE was estimated from the combined color changes of the L (lightness), a (red/green), and b (yellow/blue) values, and FI_570nm_/FI_410nm_ was defined as the ratio of the maximum emission peaks of MQ at 410 and 570 nm. ΔE was calculated according to the following equation [43]:

ΔE = [(L − L_0_)^2^ + (a − a_0_)^2^ + (b − b_0_)^2^]^1/2^ (1)

where L_0_, a_0_, and b_0_ values are the color values of H^+^MQ-loaded paper chip before treatment, while the L, a, and b values are the color values of the H^+^MQ-loaded paper chip after treatment. The fluorescence intensity of H^+^MQ-loaded paper chip was measured using a fluorescence spectrometer.

### 2.4. Stability Analysis of H^+^MQ-Loaded Paper Chips

The stability of the H^+^MQ-loaded paper chip was evaluated by recording the changes of ΔE and FI_570nm_/FI_410nm_ under different storage conditions, wherein the paper chip was stored at 25 °C, 4 °C, and −20 °C for 7, 14, and 35 days, respectively.

### 2.5. Reusability of H^+^MQ-Loaded Paper Chip

The reusability of the H^+^MQ-loaded paper chip was investigated by recording the changes of ΔE and FI_570nm_/FI_410nm_ after repeated fuming for 3 min with NH_3_ or HCl vapors. The recycle treatment with NH_3_ and HCl was repeated 10 times.

### 2.6. Freshness Monitoring by Testing BAs with H^+^MQ-Loaded Paper Chip

The weeverfish, shrimp, and chicken were purchased from a local supermarket, and were selected as real-world samples for freshness evaluation. As presented in Scheme 1c, fresh weeverfish, shrimp, and chicken were placed in the inner bottom part of sealed Petri dishes, while the as-prepared paper chips were placed in the inner top of the containers, thus effectively avoiding direct contact between the sample and the chip. Subsequently, all containers were sealed and maintained at different temperatures (−20 °C, 4 °C, and 25 °C) for the corresponding times. During the period, the changes in the color and fluorescence of the chips were recorded at regular intervals by using a phone and a fluorescence spectrometer. In addition, all samples were collected for the measurement of each biogenic amine (BAI) and total amines using the HPLC method according to the national standard method of the People’s Republic of China (GB5009.208-2016). The procedure of HPLC detection is described in the Supporting Information. All samples were determined in triplicate.

## 3. Results and Discussion

### 3.1. Characterization and Photophysical Properties of MQ

Scheme 1b shows the structural formula of MQ, in which triphenylamine and quinoxaline-2(1H)one were selected as electron-donor (D) and electron-accepting (A) units, respectively, thus creating a classical D–A structure. MQ was synthesized by Knoevenagel reactions of methylquinoxalin-2(1H)-one and 4-formyltriphenylamine [44] and was confirmed with nuclear magnetic resonance techniques in our previous publication [45]. The photophysical properties of MQ were then studied. The solvent effect of MQ was first investigated in different polar organic solvents at room temperature. Figure S1a shows that the fluorescent spectra of MQ varied with the polarity of the solvent due to the ICT effect caused by its D–A structure. The AIE effect of MQ was then investigated by collecting the UV−VIS absorption and fluorescent spectra in a mixed solution of methanol/water with different water fractions. Figure S1b,c shows that with increasing the water fraction from 0 to 80%, the fluorescent intensity at 570 nm gradually increased, and the maximum emission peak first blueshifted with the water fraction ranging from 0 to 40%, and then redshifted with the continuous increase of water fraction. This phenomenon is due to the enhanced ICT effect when increasing the solvent polarity. Notably, with further increasing the water fraction to 90%, the fluorescence emission of MQ increased obviously, which is attributed to the formation of aggregates in high water fractions, thus resulting in the restriction of intramolecular motion to activate the AIE effect. These results indicate that MQ has typical AIE and ICT characteristics.

Previous research has demonstrated that the optical properties of the D–A structure molecules are tunable by protonation on the donor/acceptor to alter the D–A interaction [33,46], thus causing a blue/redshifted emission. Considering this, we investigated the effect of the donor/acceptor protonation on the UV−VIS absorption and fluorescent spectra of MQ. As shown in Scheme 1b, MQ first reacted with HCl to form the protonated MQ (i.e., H^+^MQ). With the donor protonation of MQ, the maximum absorption and emission peaks at 446 nm and 606 nm moved to 590 nm and 490 nm (Figure S1d,e), respectively. The results are consistent with the fluorescent variations of MQ against the pH values (Figure S1f), thus providing a basis for biogenic amine detection. The possible reason is that the protonation of the imine unit from the triphenylamine donor weakened the D–A interaction, thus activating the ICT effect of MQ.

### 3.2. Preparation and Characterization of the H^+^MQ-Loaded Paper Chip

**Figure 1:**
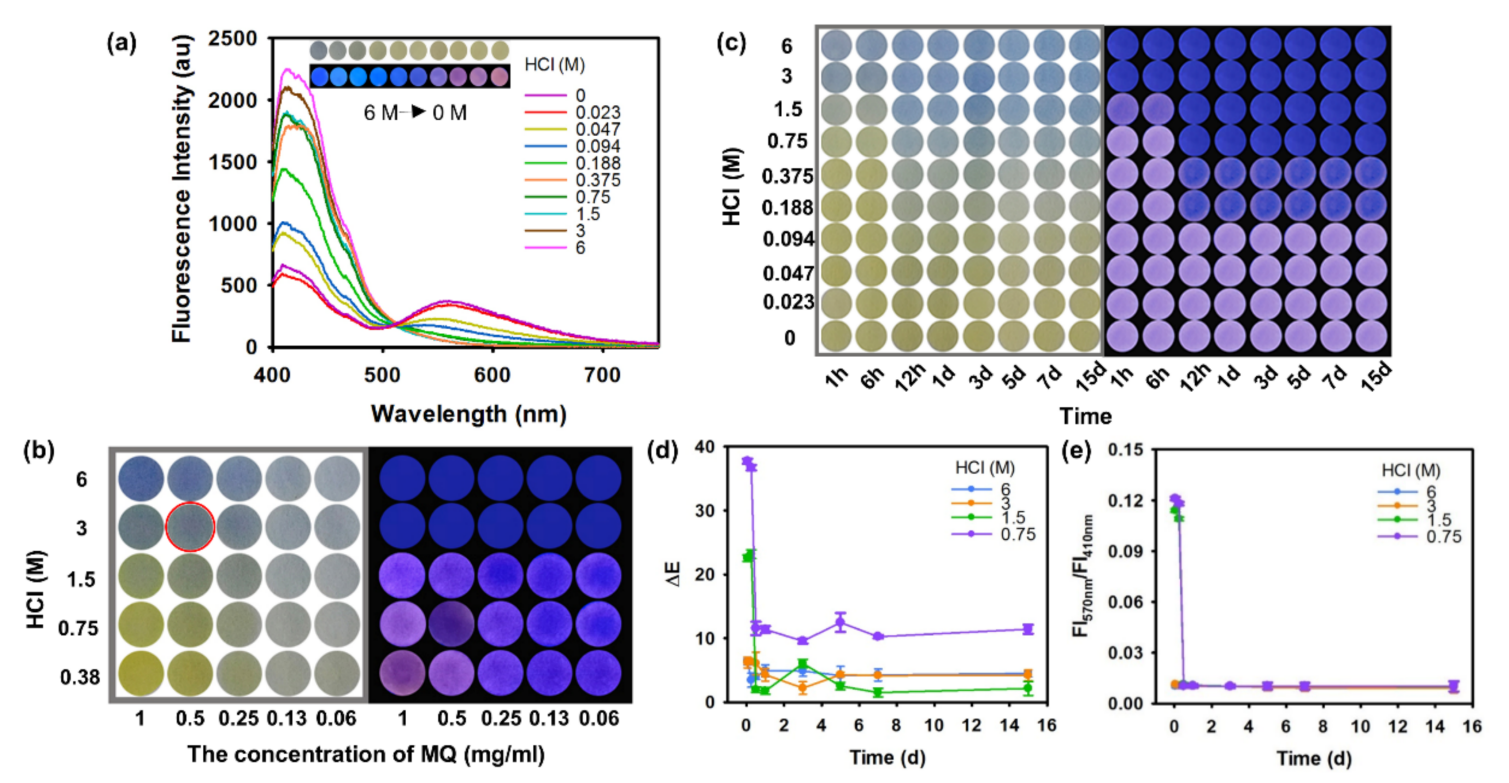
(**a**) Photoluminescence spectra of the H^+^MQ-loaded paper chip in response to different HCl concentrations. (**b**) Optimization of HCl and MQ deposited on the paper chip. (**c**) Photographs, (**d**) DE, and (**e**) FI_570nm_/FI_410nm_ of the H^+^MQ-loaded paper chip when treated with 5% glycerinum and 5% sucrose during storage.

MQ is almost non-emissive in solution but shows high-efficiency luminescence in aggregated or solid state, which makes it have a great application potential in solid-phase support. Therefore, the H^+^MQ-loaded paper chip was prepared to investigate the capability of the system for sensing amine vapors in the solid state. Qualitative filter papers were chosen as solid phase carriers because of their porous structures and excellent loading and adsorption capacity. The MQ-loaded paper chip was first prepared by depositing MQ solution on the filter paper. The MQ-deposited paper chip was then treated with different concentrations of HCl ranging from 0 to 6 M. The results in Figure 1a show that the MQ-deposited paper chip has similar pH responses in color and fluorescence to those for the MQ solution. The photoluminescence spectra of the MQ-loaded paper chip showed that the fluorescence intensity at 410 nm (FI_410__nm_) increased gradually with the increase of HCl concentration from 0 to 6 M, while the fluorescence intensity at 570 nm (FI_570__nm_) decreased. These results indicate that the loading of MQ on the filter paper would not affect its pH-response optical properties, and the prepared MQ-loaded paper chip could be used as a ratiometric and colorimetric indicator to detect amine vapors. To achieve the obvious color change and the best amine response, the concentrations of MQ and HCl for preparing the H^+^MQ-loaded paper chip were optimized using a similar checkerboard titration method under different MQ concentrations of 0.06–1 mg/mL with changing HCl concentrations from 0.38 to 6 M. Figure 1b shows that the optimal concentration combinations for MQ and HCl were 0.5 mg/mL and 3 M, respectively. Under the developed condition, the storage stability of the H^+^MQ-loaded paper chip was investigated by treating this paper chip with different concentrations of HCl, as it is essential for long-term food freshness monitoring. Figure S2a revealed the color and fluorescence changes of the H^+^MQ-loaded paper chip within 15 days of storage. The results showed the color of the H^+^MQ-loaded paper chip changed to yellow with the fluorescence shifting to amaranth after 5 days of storage under all HCl-treated concentrations, which was further verified by the variations of ΔE and FI_570nm_/FI_410nm_ ( Figure S2b,c). Several possible reasons are responsible for these phenomena: (i) the protonation of MQ by HCl takes a certain time due to the diffusion and penetration of acid on the paper chip; (ii) the qualitative filter paper has a limited loading content of HCl; and (iii) the formed H^+^MQ suffers the risk of deprotonation during paper chip storage because of the volatility of HCl, thus resulting in the invalidity of the paper chip for the ammonia response.

To improve the long-term storage stability of the prepared H^+^MQ-loaded paper chip, an HCl solution containing 5% glycerinum and 5% sucrose was suggested to treat the MQ-loaded filter paper, as this could increase the acid loading content and reduce the volatilization of HCl. As can be seen from Figure 1c, when the concentration of HCl was greater than 3 M, the color and fluorescence changes of the obtained H^+^MQ-loaded paper chip were negligible during the storage period of 15 days. Figure 1d,e shows that the ΔE and FI_570nm_/FI_410nm_ of the obtained paper chip decreased sharply first with HCl treatment, and then reached a constant during the observation period. These results indicated that the H^+^MQ-loaded paper chip has a good storage stability. Subsequently, we further investigated the stability of the MQ-loaded paper chip stored at 25 °C, 4 °C, and −20 °C, respectively. Figure S3 shows that no obvious changes were observed for the L, a, and b values when the paper chips were stored at 25 °C for 7 days, 4 °C for 14 days, and −20 °C for 35 days. The above results indicated the applicability of paper chips for long-term real-time freshness monitoring at different temperatures.

**Figure 2:**
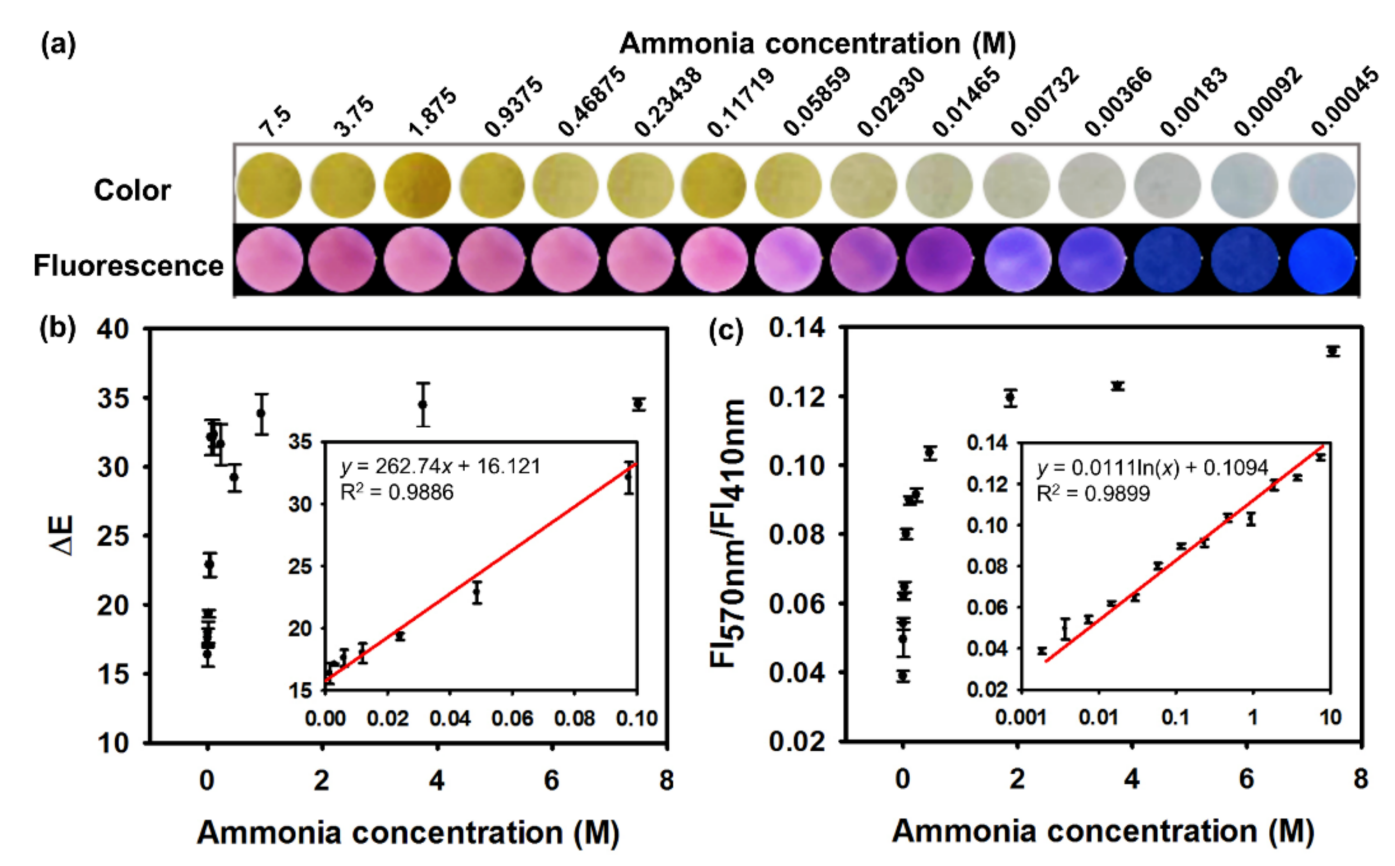
The color and fluorescence changes of the H^+^MQ-loaded paper chip in response to varied concentrations of ammonia. (**a**) Photographs, (**b**) ΔE, and (**c**) FI_570nm_/FI_410nm_ of the reacted paper chips under different ammonia concentrations.

Encouragingly, the ammonia response behavior of the H^+^MQ-loaded paper chip was studied by exposing it to different concentrations of ammonia vapor, ranging from 0.00045 to 7.5 M. Figure 2a exhibits the changes of color and fluorescence of the H^+^MQ-loaded paper chip with an ammonia concentration. With the increase of ammonia concentration from 0.00045 to 7.5 M, the color of the paper chip changed from blue to yellow, and the fluorescence changed from blue to amaranth. For colorimetric detection, the change of ΔE to ammonia concentration is shown in Figure 2b. The ΔE of the paper chip increased gradually with the increase of ammonia concentration, and there was a good linear relationship between them in the ammonia concentration range of 0.00183–0.05859 M (R^2^ = 0.9886). The detection limit (LOD) for colorimetric response was 0.00107 M according to the mean plus three-fold standard deviations of 20 negative samples. For fluorescent detection, Figure S4 shows the fluorescent spectra of the MQ-loaded paper chip after exposure to different concentrations of ammonia vapor. With the increase in ammonia concentration, the emission peak at 410 nm gradually decreased, and that at 570 nm gradually increased. Consequently, the variation of FI_570nm_/FI_410nm_ to ammonia concentration is displayed in Figure 2c, wherein FI_570nm_/FI_410nm_ increased with the increase of ammonia concentration, and an excellent linearity was obtained between FI_570nm_/FI_410nm_ and the logarithm of ammonia concentration ranging from 0.00183 to 7.5 M (R^2^ = 0.9899). The LOD for the fluorescence response was 0.00180 M. These results indicate that the prepared MQ-loaded paper chip is suitable for sensitive freshness monitoring by measuring its color and fluorescence response to ammonia.

The reversibility and reusability were further tested by alternately exposing the MQ-loaded paper chip to ammonia and HCl for multiple cycles. As shown in Figure S5, when paper chips were exposed to ammonia vapor, their color turned yellow, and their fluorescence changed from blue to amaranth. On the contrary, when paper chips were moved to the HCl atmosphere, the color and fluorescence of the paper chip returned to the original state after being fumigated by HCl. More importantly, the constructed paper chips exhibited negligible changes in the color and fluorescence, even after ten cycles. The reason for its reversibility is that the protonation and deprotonation of MQ are essentially reversible, thus providing an opportunity for reversible regulation of its optical properties. The results implied that the H^+^MQ-loaded paper chip has a good stability, ammonia reactivity, reversibility, and reusability, and has a good application prospect in developing a pH-response sensing system for food freshness monitoring.

### 3.3. Monitoring of Meat and Seafood Freshness

Encouraged by the above results, we further verified the feasibility of testing ammonia vapors from spoiled meat and seafood. To obtain the best sensitivity of H^+^MQ-loaded paper chips, the weights of meat and seafood for freshness monitoring were first optimized through determining the ammonia vapors generated from different weights of chicken, weeverfish, and shrimp (10, 20, 30, 40, and 50 g) at different time points using the prepared H^+^MQ-loaded paper chip at room temperature. Figure S6a–c reveals that the color of paper chips varied from blue to yellow over time at all given weights of chicken, weeverfish, and shrimp. Notably, the color response time of the H^+^MQ -loaded paper chips was shorter as the weight of meat and seafood increased, which is due to the production of more BAs. The corresponding ΔE variations against weight and time are shown in Figure S6d–f. The results show that the ΔE values of the paper chips were the highest when the weight of the meat and seafood was 40 g, suggesting that 40 g was the optimal weight for meat and seafood freshness monitoring.

**Figure 3:**
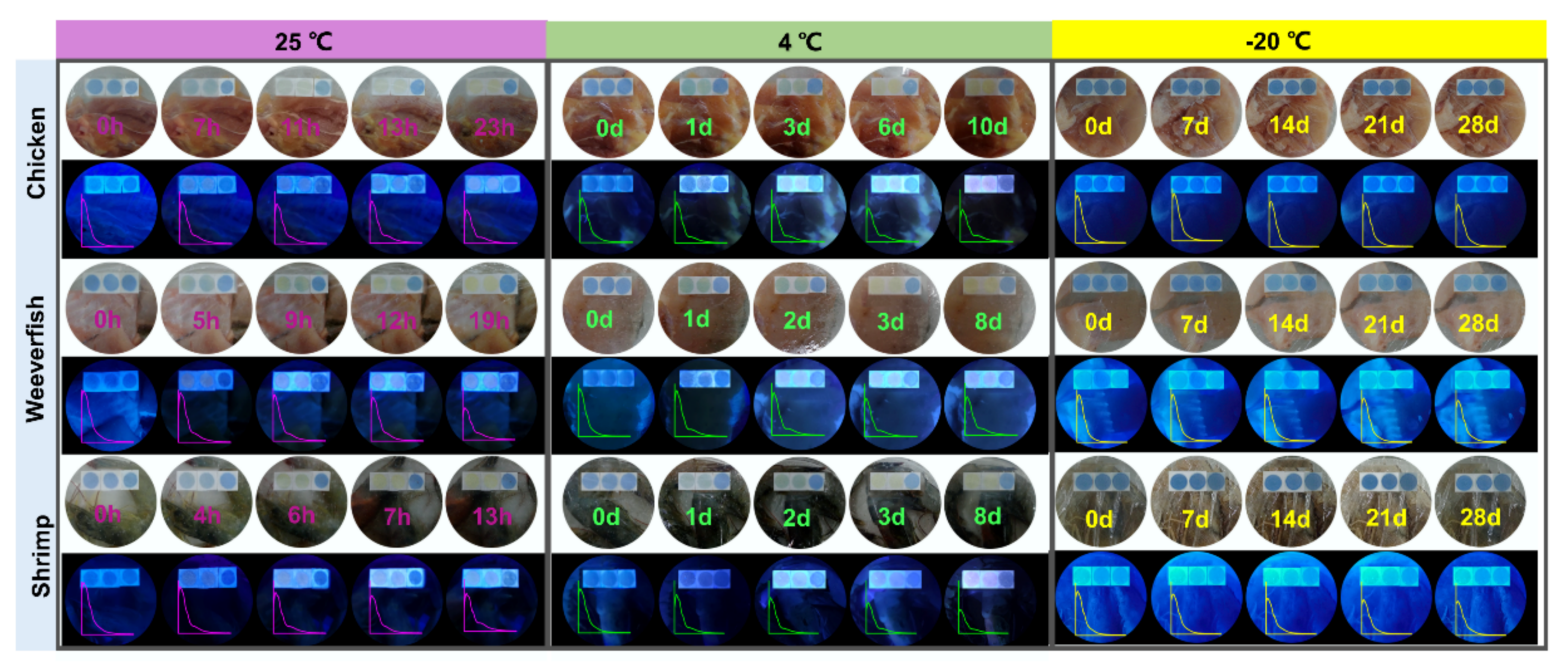
Real-time and visual monitoring of BAs produced from chicken, weeverfish, and shrimp at different storage temperatures of 25 °C, 4 °C, and −20 °C. The first two paper chips in each dish indicate the experimental group and the last paper chip indicates the control group.

Under the optimal condition, the prepared H^+^MQ-loaded paper chips were then employed as an indicator to monitor food freshness. Fresh chicken, weeverfishs, and shrimp (40 g) were put in sealed Petri dishes, and the paper chips were put in the inner top to monitor the production of ammonia vapors under different storage temperatures. The prototypes and corresponding fluorescence images of the paper chips under daylight and ultraviolet light are summarized in Figure 3. The results indicate that with the increase of storage temperature from −20 °C to 25 °C, the time for producing a distinct color and fluorescence change of the paper chips was gradually shortened. Meat and seafood samples were stored at 4 °C for 1 day, and obvious color and fluorescence responses were observed, while when stored at 25 °C they only required 5–7 h. Notably, when stored at −20 °C, no significant changes were observed. These findings suggest that a high storage temperature is beneficial to the production of BAs from meat and seafood samples, thus triggering the corresponding color and fluorescence changes of the H^+^MQ indicator due to its protonation. Subsequently, we further collected ΔE and FI_570nm_/FI_410nm_ variations and the results are summarized in Tables S1–S9 for the succeeding analysis.

**Figure 4:**
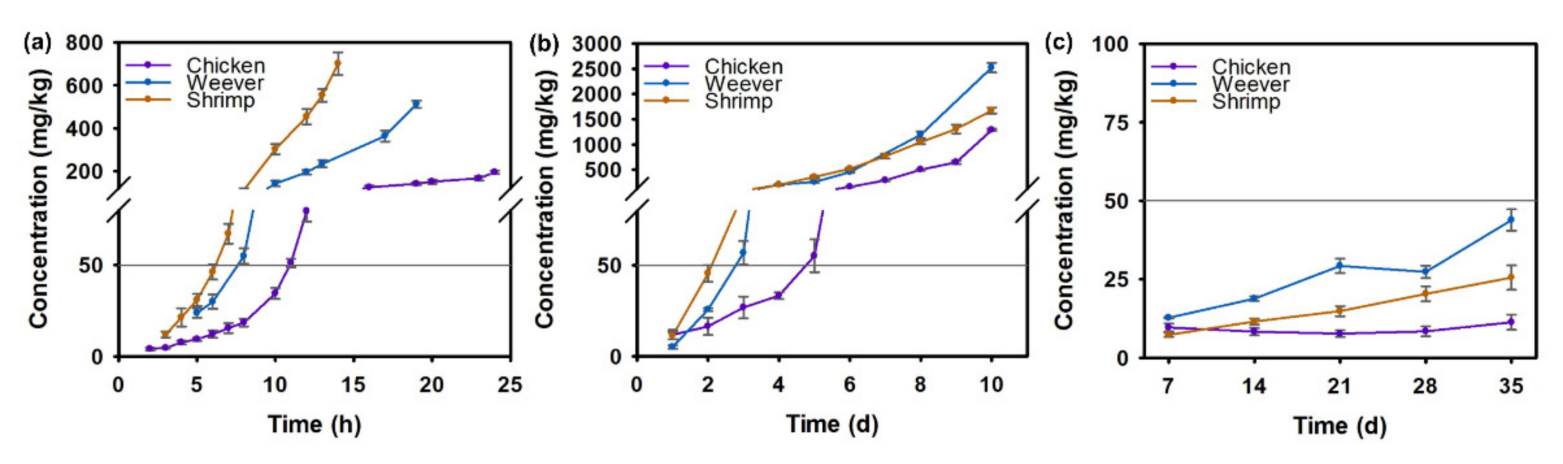
Real-time BAI monitoring of chicken, weeverfish, and shrimp at different storage temperatures using the HPLC method: (**a**) 25 °C, (**b**) 4 °C, and (**c**) −20 °C. The red dashed line shows the BAI concentration of 50 mg/kg, which indicates spoilage.

HPLC has a high sensitivity and reliability, which is widely regarded as a reference method for the determination of BAs in the spoilage of food [47,48]. Therefore, for comparison, we selected HPLC to simultaneously monitor biogenic amine production. A key indicator, BAI, which is the sum (mg/kg) of the concentrations of putrescine, cadaverine, histamine, and tyramine, was used to evaluate the meat and seafood quality, wherein BAI > 50 mg/kg indicates spoilage [49,50]. The species and content of BAs in chicken, weeverfish, and shrimp at different storage temperatures (25 °C, 4 °C, and −20 °C) were determined and quantified by the HPLC method (Figures S7 and S8), and the corresponding results are shown in Figure 4 and Tables S1–S9. The real-time color and fluorescence changes of the H^+^MQ-loaded paper chips were recorded (Figure 3), and the corresponding ΔE and FI_570nm_/FI_410nm_ are summarized in Table S1–S9. As shown in Figure 3, the H^+^MQ-loaded paper chips showed obvious color and fluorescence changes with the increase in storage time, which can be clearly distinguished by the naked eye compared with the initial color and fluorescence. The paper chips changed to yellow from the initial blue with ΔE of 23.17 ± 2.10 and FI_570nm_/FI_410nm_ of 0.078 ± 0.002 after 11 h (Table S1). At this point, the BAI value of the chicken increased from 0 mg/kg to 51.23 ± 2.32 mg/kg, indicating that the chicken had spoiled. With further extending the storage time, the BAI value continued to increase, thus resulting in further increases of ΔE and FI_570nm_/FI_410nm_. Similar results were observed for weeverfish and shrimp for storage at 25 °C; however, a shorter storage time of 8 h for weeverfish (Table S2) and 7 h for shrimp (Table S3) could result in a BAI value over 50 mg/kg, suggesting the spoilage of weeverfish and shrimp samples. At this point, the ΔE and FI_570nm_/FI_410nm_ of paper chips were 28.90 ± 1.24 and 0.085 ± 0.003 for weeverfish, and 25.36 ± 0.91 and 0.081 ± 0.004 for shrimp. When fresh samples of chicken, weeverfish, and shrimp were stored at 4 °C for 5, 3 and 3 days, the original blue of the paper chip changed to yellow with the fluorescence changing from blue to amaranth (Figure 3). As can be seen from Tables S4–S6, the ΔE values for chicken, weeverfish, and shrimp were 32.65 ± 0.64, 29.63 ± 2.15, and 30.66 ± 2.13, respectively, and the FI_570nm_/FI_410nm_ values were 0.114 ± 0.003, 0.091 ± 0.002, and 0.106 ± 0.004, respectively. At these time points, the BAI values of the three samples were higher than 50 mg/kg, indicating that the meat and seafood had spoiled. After being kept at −20 °C for 35 days, the BAI values of the three food samples were less than 50 mg/kg (Tables S7–S9). The color and fluorescence of the corresponding paper chips hardly changed (Figure 3), thus leading to ΔE and FI_570nm_/FI_410nm_ values less than 10.10 and 0.013, respectively. These results indicate that the chicken, weeverfish, and shrimp were still fresh. Notably, the color and fluorescence changes of the chicken were slower than those of the weeverfish and shrimp, indicating that the freshness of chicken was longer than those of weeverfish and shrimp under the same storage conditions. In addition, the ΔE and FI_570nm_/FI_410nm_ of paper chips used for spoilage indication are different among the three samples (Tables S1–S9), which may be attributed to differences in the species and amounts of BAs produced by different foods [51] and microorganisms. Taken together, these results confirm that the fabricated H^+^MQ-loaded paper chip can act as an effective indicator for real-time and nondestructive monitoring of packaged food freshness. Moreover, Table S10 provided an overview about the performance of the proposed sensing system compared with previously reported methods for BAs detection.

## 4. Conclusions

In conclusion, we successfully developed a paper-based smart sensor chip by depositing H^+^MQ on the qualitative filter paper using the physical deposition method. The prepared paper chip showed an excellent bimodal colorimetric and fluorescence response to amine vapors based on the deprotonation of H^+^MQ. Using this characteristic, H^+^MQ was used as amine-response indicators for the sensitive detection of biogenic amine contents in three different meat and seafood products at different storage temperatures. The H^+^MQ-loaded paper chip can effectively distinguish the food freshness based on its color difference and the ratio of fluorescence change. The relationship between the ΔE and FI_570nm_/FI_410nm_ of paper chips and food freshness was constructed by simultaneously measuring the biogenic amine contents in packaged food using HPLC and the developed method. The ΔE and FI_570nm_/FI_410nm_ of paper chips increased with the increase in spoilage degree, showing the feasibility of the designed H^+^MQ-loaded paper chip for evaluating the food spoilage degree. Thus, this work provides a promising smart sensor chip using a pH stimuli AIEgen as bimodal indicators, which can be easily applied to sensitive detection of food spoilage for real-time, visual, and non-destructive monitoring of food freshness.

## Data Availability

Data is contained within the article or supplementary material.

## Supplementary Materials

The following are available online at https://www.mdpi.com/article/10.3390/foods11070932/s1. Figure S1: The photophysical properties of MQ. Figure S2: The stability of H+MQ-loaded paper chip treated with different concentration of HCl during the storage. Figure S3: The stability of H^+^MQ-loaded paper chip at different storage temperatures. Figure S4: Photoluminescence spectra of H^+^MQ-loaded paper chip in response to different concentrations of ammonia. Figure S5: The reversibility evaluation of H^+^MQ-loaded paper chip by alternately exposing it to ammonia (0.03 M) and HCl (3 M) for ten cycles. Figure S6: Optimization of the food weights (10, 20, 30, 40 and 50 g) for biogenic amine monitoring. Figure S7: HPLC chromatograms of mixed standard BAs of different concentration. Figure S8: Standard curves of nine standard BAs with peak areas against the concentrations of standard BAs. Table S1: Changes of BAs in chicken during storage and corresponding ΔE and FI_570nm_/FI_410nm_ of H^+^MQ-loaded paper chip. Table S2: Changes of BAs in weeverfish during storage and corresponding ΔE and FI_570nm_/FI_410nm_ of H^+^MQ-loaded paper chip. Table S3: Changes of BAs in shrimp during storage and corresponding ΔE and FI_570nm_/FI_410nm_ of H^+^MQ-loaded paper chip. Table S4: Changes of BAs in chicken during storage and corresponding ΔE and FI_570nm_/FI_410nm_ of H^+^MQ-loaded paper chip. Table S5: Changes of BAs in weeverfish during storage and corresponding ΔE and FI_570nm_/FI_410nm_ of H^+^MQ-loaded paper chip. Table S6: Changes of BAs in shrimp during storage and corresponding ΔE and FI_570nm_/FI_410nm_ of H^+^MQ-loaded paper chip. Table S7: Changes of BAs in chicken during storage and corresponding ΔE and FI_570nm_/FI_410nm_ of H^+^MQ-loaded paper chip. Table S8: Changes of BAs in weeverfish during storage and corresponding ΔE and FI_570nm_/FI_410nm_ of H^+^MQ-loaded paper chip. Table S9: Changes of BAs in shrimp during storage and corresponding ΔE and FI_570nm_/FI_410nm_ of H^+^MQ-loaded paper chip. Table S10: Comparison of the proposed sensing system with other reported methods for BAs detection. References [46,52,53,54,55,56,57] are cited in the Table S10.
