# Supplementary material for: Ratiometric Monitoring of Biogenic Amines by a Simple Ammonia-Response Aiegen
Source: Foods. 2022 Mar 24;11(7):932. doi: 10.3390/foods11070932 (PMC8997827; doi:10.3390/foods11070932)
Supplement: Supplementary file 1 [file foods-11-00932-s001.zip › foods-1631250-supplementary.pdf]

Supporting Information for:

Ratiometric monitoring of biogenic amines by a simple ammonia-response AIEgen

Xujing Guo[1,2], Xirui Chen[1], Rui Chen[3,*], Yujie Tu[4], Tianying Lu[1], Yuqian Guo[1], Liang Guo[2], Yonghua Xiong[1,2], Xiaolin Huang[1*], and Ben Zhong Tang[4,5]

1 State Key Laboratory of Food Science and Technology, School of Food Science and Technology, Nanchang University, Nanchang 330031, P. R. China
2 Jiangxi-OAI Joint Research Institute, Nanchang University, Nanchang 330047, P. R. China
3 Key Laboratory of Clinical Laboratory Diagnostics (Ministry of Education), College of Laboratory Medicine, Chongqing Medical University, Chongqing, 400016, China
4 AIE Institute, Guangzhou Development District, Huangpu, Guangzhou, 510530, P. R. China
5 Shenzhen Institute of Aggregate Science and Technology, School of Science and Engineering, The Chinese University of Hong Kong, Shenzhen, Guangdong 518172, China
* Correspondence: SCCR1991@163.com (R.C.); hxl19880503@163.com (X. H.)

Procedure of HPLC detection

Preparation of standard BAs solutions

Hydrochloric acid (HCl) (0.1 M) solutions of nine standard BAs were mixed with the same volume of BAs (tryptamine, phenylethylamine, putrescine, cadaverine, histamine, octopamine, tyramine, spermidine, spermine) at different concentrations (0.78, 1.56, 3.125, 6.25, and 12.5 mg/L). The as-prepared sample solutions were used for subsequent measurement.

Preparation of sample solutions

The procedure of amines extraction was carried out following the national standard method of the People's Republic of China (GB5009.208-2016). Each sample (chicken, weeverfish or shrimp) was minced. 10 g of minces were homogenized with 500 μL of 0.1 M HCl solutions containing 100 mg/L 1,7-diaminoheptane. The homogeneous sample solution was then mixed with 20 mL of 5% trifluoroacetic acid (TCA) for 30 min under violent vertexing. After centrifugation at 5000 rpm for 10 min, the residue was re-extracted with the same procedure above. The two supernatant solutions were collected, combined, and diluted to the final volume of 50 mL with 5% TCA.

0.5 g of sodium chloride (NaCl) was added to 10 mL of the above extraction solution. After the complete dissolution of NaCl under vigorous vertexing, 10 mL of *n*-hexane was added to the mixed solution for another 5 min vertexing. The mixed solution was then stand and layered. After discarding the upper organic phase, the lower layer of solution was degreased again with the same procedure above.

5 mL of the above extraction solution was adjusted pH to 12 with 5 mol/L sodium hydroxide (NaOH) solution, followed the addition of 5 mL of *n*-butyl alcohol/chloroform solution with the volume ratio of 1:1. After vigorous vertexing for 5 min, the solution was then centrifuged at 5000 rpm for 5 min. The supernatants were extracted again with the same procedure above. The two precipitates were collected and diluted to the final volume of 10 mL with *n*-butyl alcohol/chloroform (1:1) solution. 5 mL of the extracted solution was mixed with

200 μL of 1 M HCl solution, and the mixture solution was dried with nitrogen in a water bath at 40 °C. Finally, the dried residue was dissolved in 1 mL of 0.1 M HCl solution for derivatization.

Derivatization procedure

The as-prepared HCl resuspension was mixed with 1 mL of saturated sodium bicarbonate solution, 100 μL of 1 M NaOH solution, and 1 mL of 10 mg/mL acetonic solution of dansylchloride. The mixed solution was placed in a water bath at 60 °C for 15 min, followed by the addition of 100 μL of 50 mg/mL sodium glutamate solution for reaction another 15 min at 60 °C. After the solution was cooled, 1 mL of water was added to remove the residual acetone by blowing nitrogen in a water bath at 40 °C. Subsequently, 0.5 g of NaCl was added to the above solution. After NaCl was completely dissolved, 5 mL of ethyl ether was added for 2 min vigorous vertexing. The mixed solution was then stand and layered. The lower aqueous phase was extracted again with the same procedure above. The two upper organic phases were collected, combined, and dried with nitrogen in a water bath at 40 °C. The dried residue was dissolved in 1 mL of acetonitrile solution and the mixed solution was filtered through a 0.22 μm microfiltration membrane filter.

Chromatographic conditions

Samples were measured by HPLC. HPLC separation was performed on a Grace Smart C 18 column. The mobile phases included 90% acetonitrile/10% (0.01 M ammonium acetate solution containing 0.1% acetic acid; solvent A), and 10% acetonitrile/90% (0.01 M ammonium acetate solution containing 0.1% acetic 10% acid; solvent B), for flow rate of 0.8 mL/min at 35 °C.

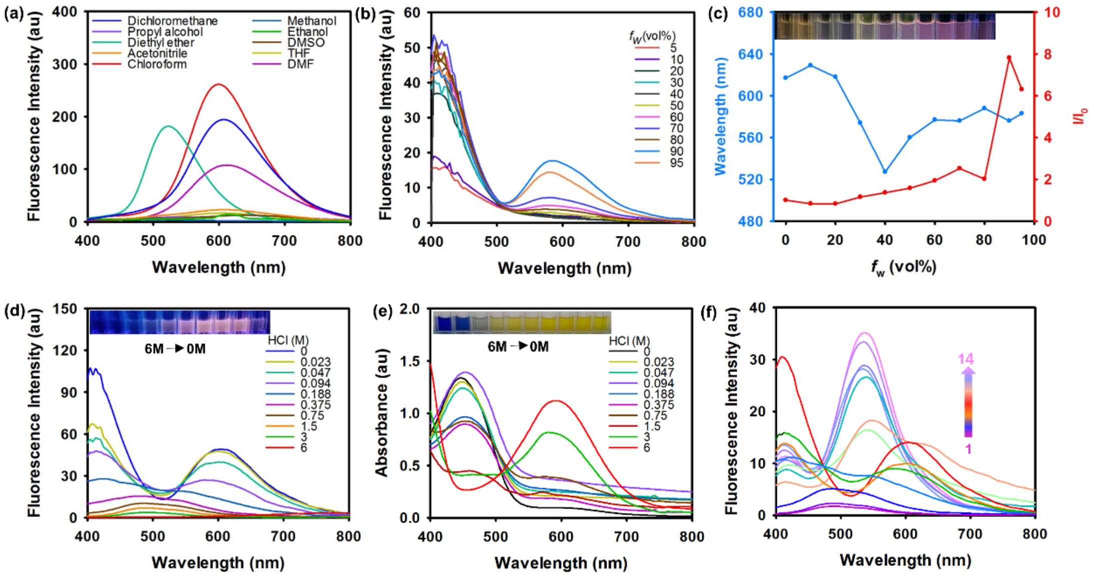

Figure S1. The photophysical properties of MQ. (a) Photoluminescence spectra of MQ in different solvents. (b) Photoluminescence spectra of MQ in MeOH/water mixtures with different water fractions (f_w). (c) The plot of I/I_0 (red line) and peak emission wavelength (blue line) versus f_w (I_0 = intensity at f_w = 0). (d) Photoluminescence spectra and (e) UV–vis spectra of MQ in different concentration of HCl (6 M-0 M). (f) Photoluminescence spectra of MQ in different pH solutions.

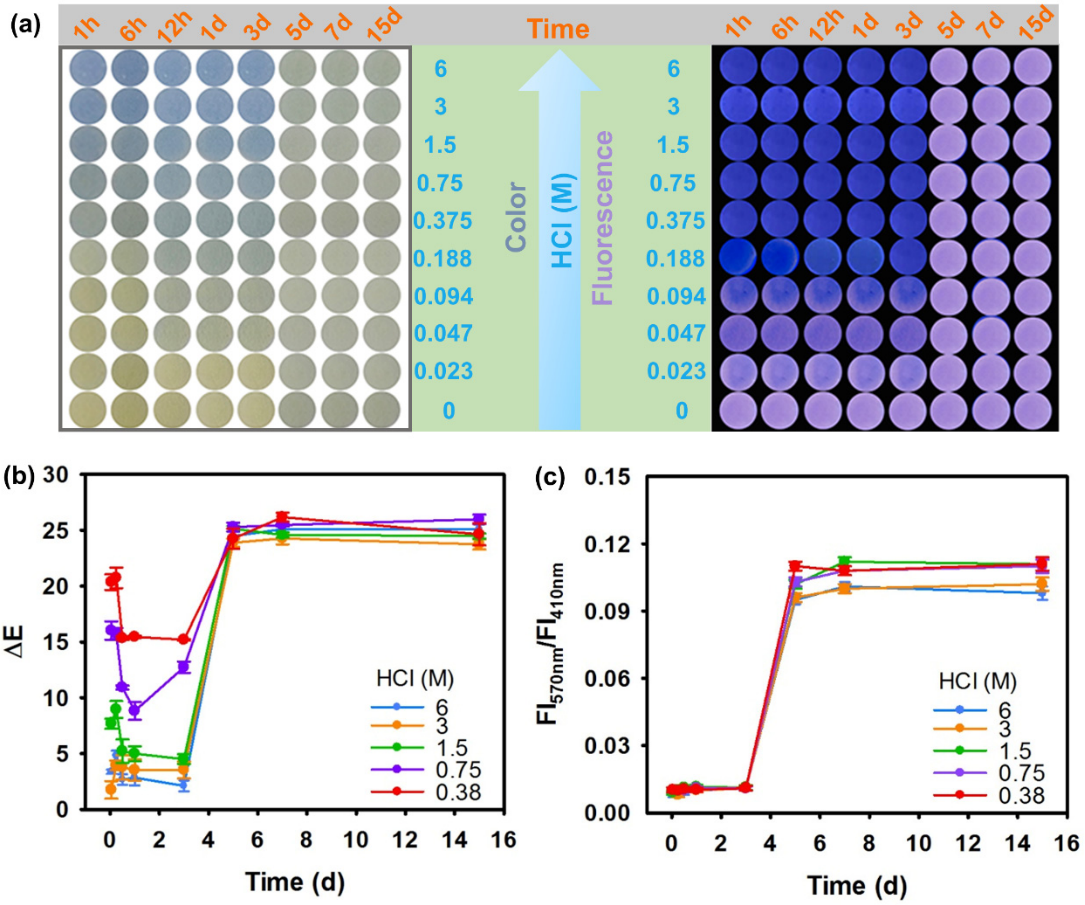

Figure S2. The stability of H^+MQ-loaded paper chip treated with different concentration of HCl during the storage. (a) Photographs, (b) ΔE, and (c) FI_{570nm}/FI_{410nm} of the reacted paper chips.

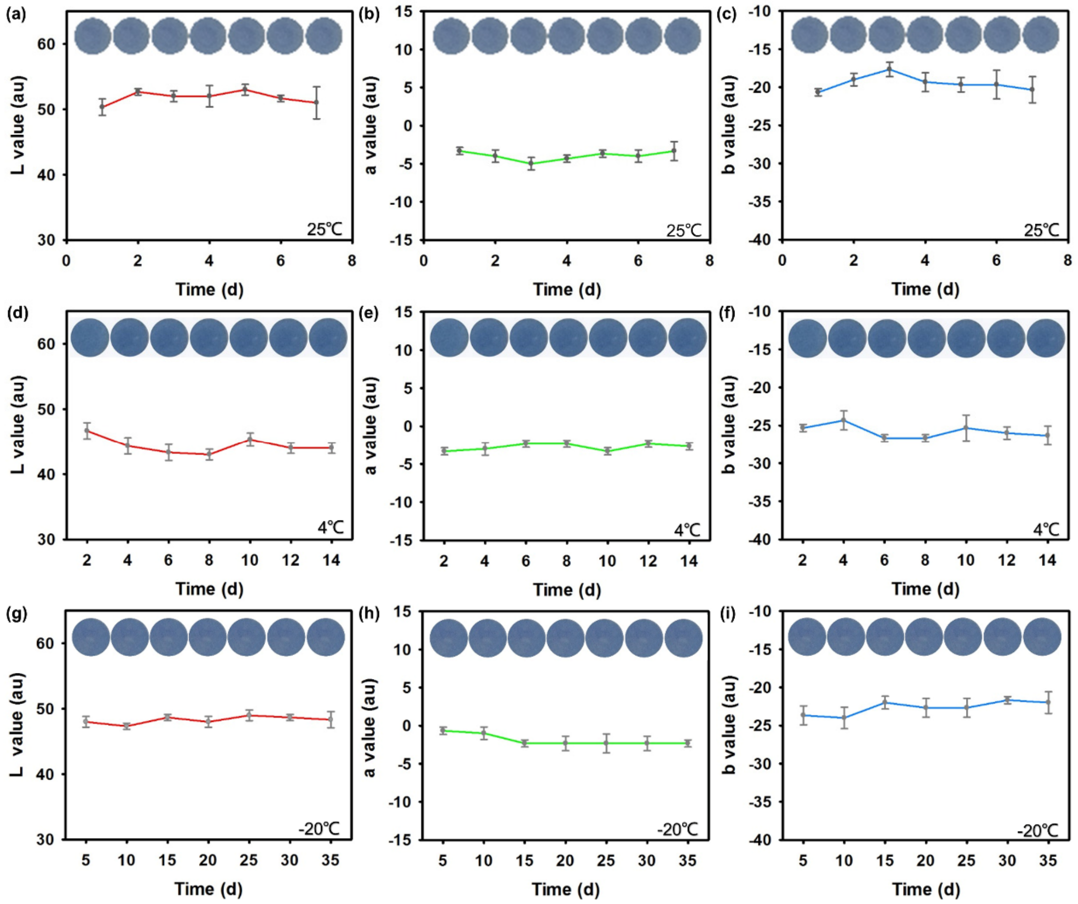

Figure S3. The stability of H+MQ-loaded paper chip at different storage temperatures. The L, a, and b vale at (a-c) 25 ℃, (d-f) 4 ℃, and (g-i) -20 ℃.

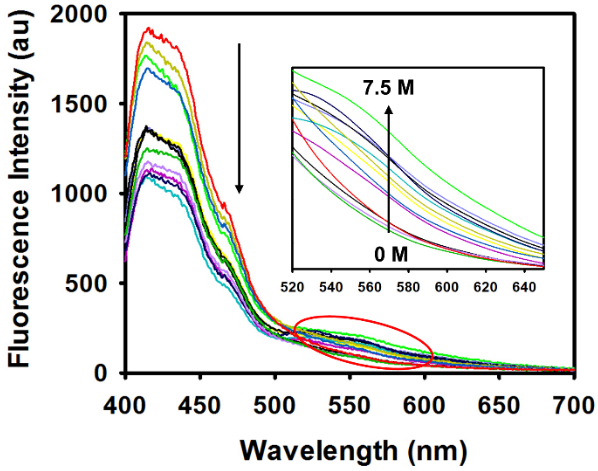

Figure S4. Photoluminescence spectra of H+MQ-loaded paper chip in response to different concentrations of ammonia ranged from 0 to 7.5 M.

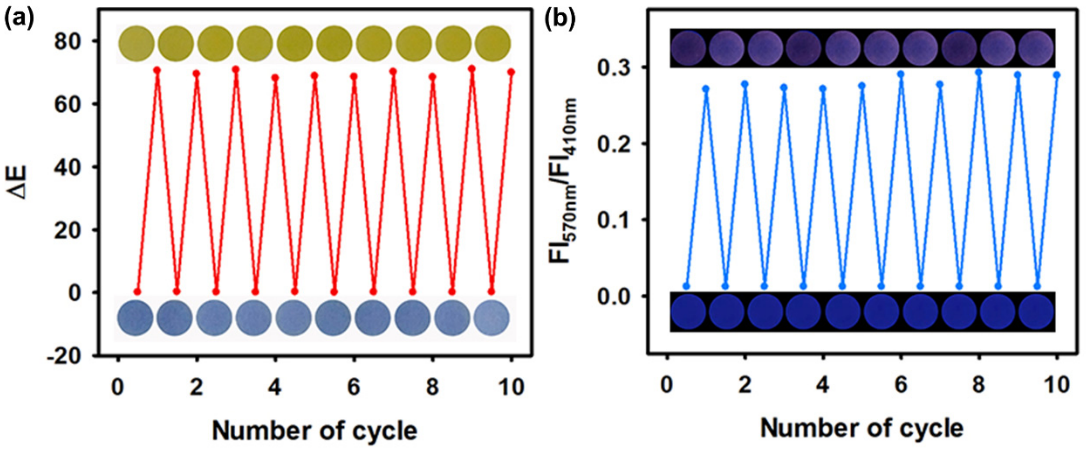

Figure S5. The reversibility evaluation of H^+MQ-loaded paper chip by alternately exposing it to ammonia (0.03 M) and HCl (3 M) for ten cycles. (a) ΔE and (b) FI_{570nm}/FI_{410nm}.

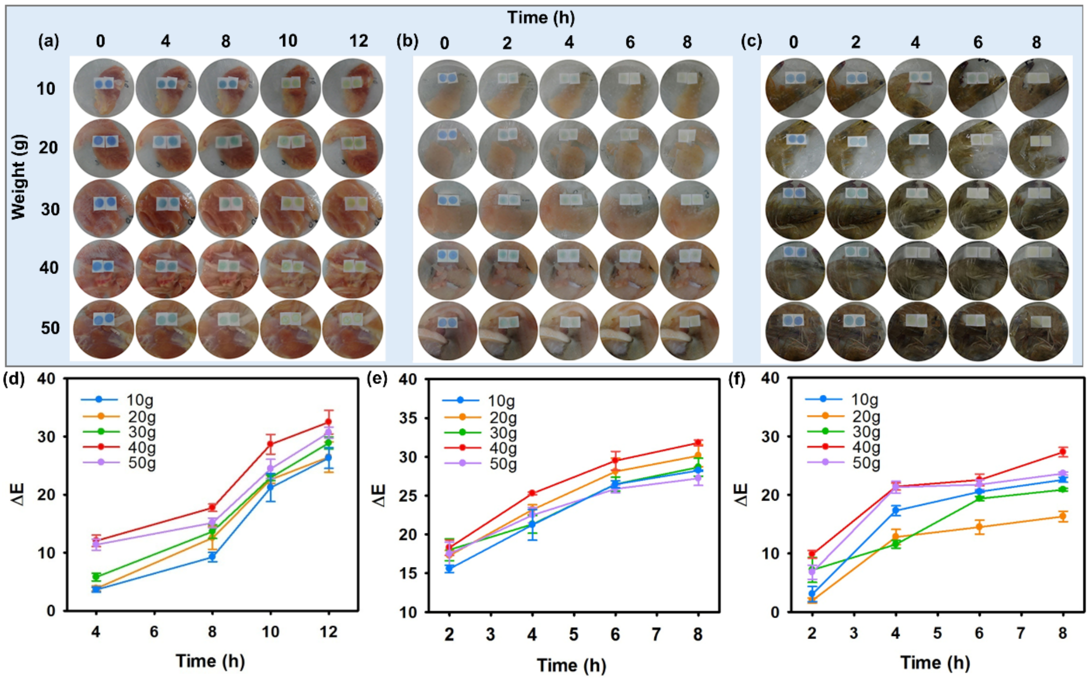

Figure S6. Optimization of the food weights (10, 20, 30, 40 and 50 g) for biogenic amine monitoring. The photographs and the corresponding ΔE value of BAs monitoring of (**a** and **d**) chicken, (**b** and **e**) weeverfish, and (**c** and **f**) shrimp at different weights.

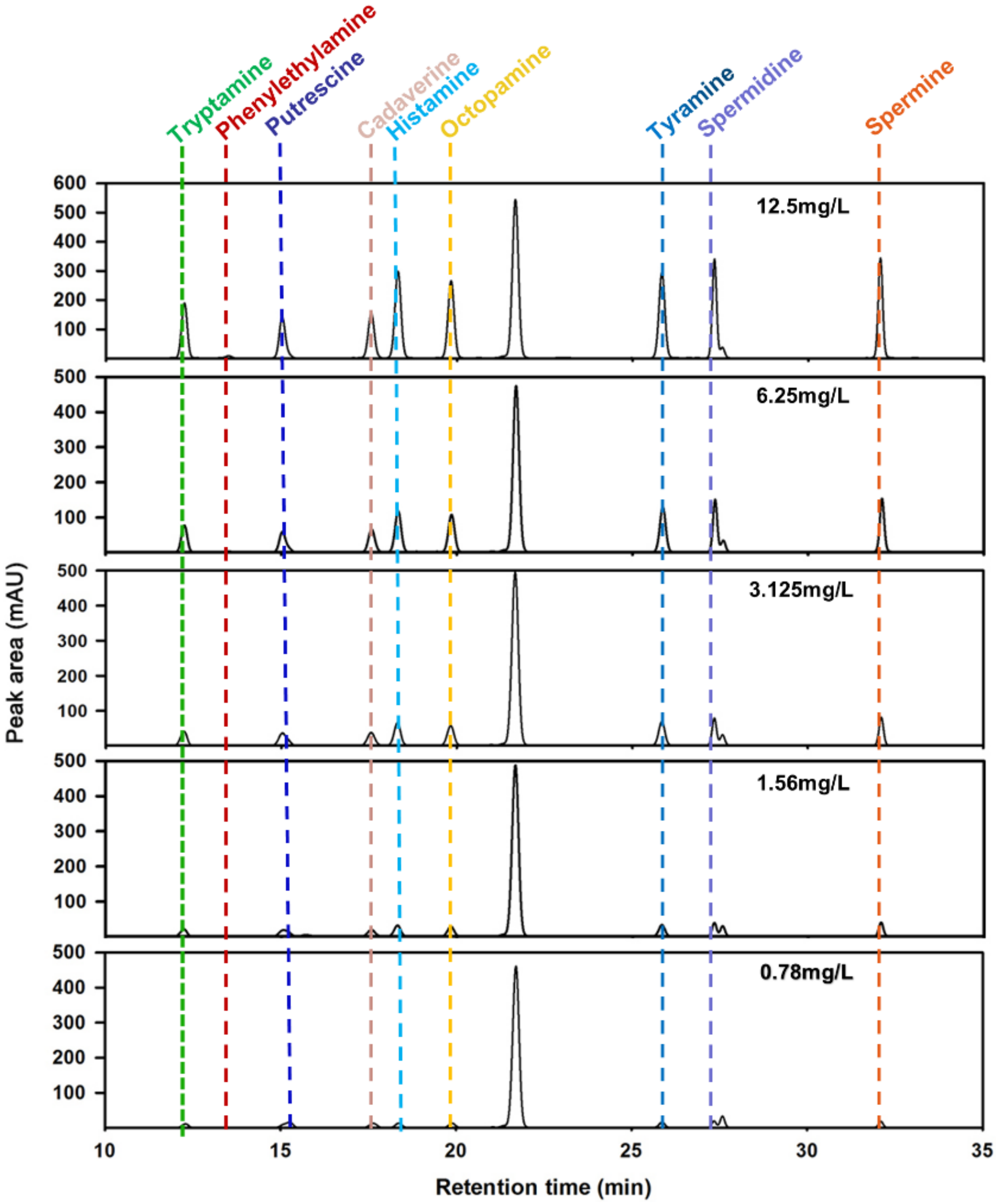

Figure S7. HPLC chromatograms of mixed standard BAs of different concentration (12.5, 6.25, 3.125, 1.56, and 0.78 mg/L).

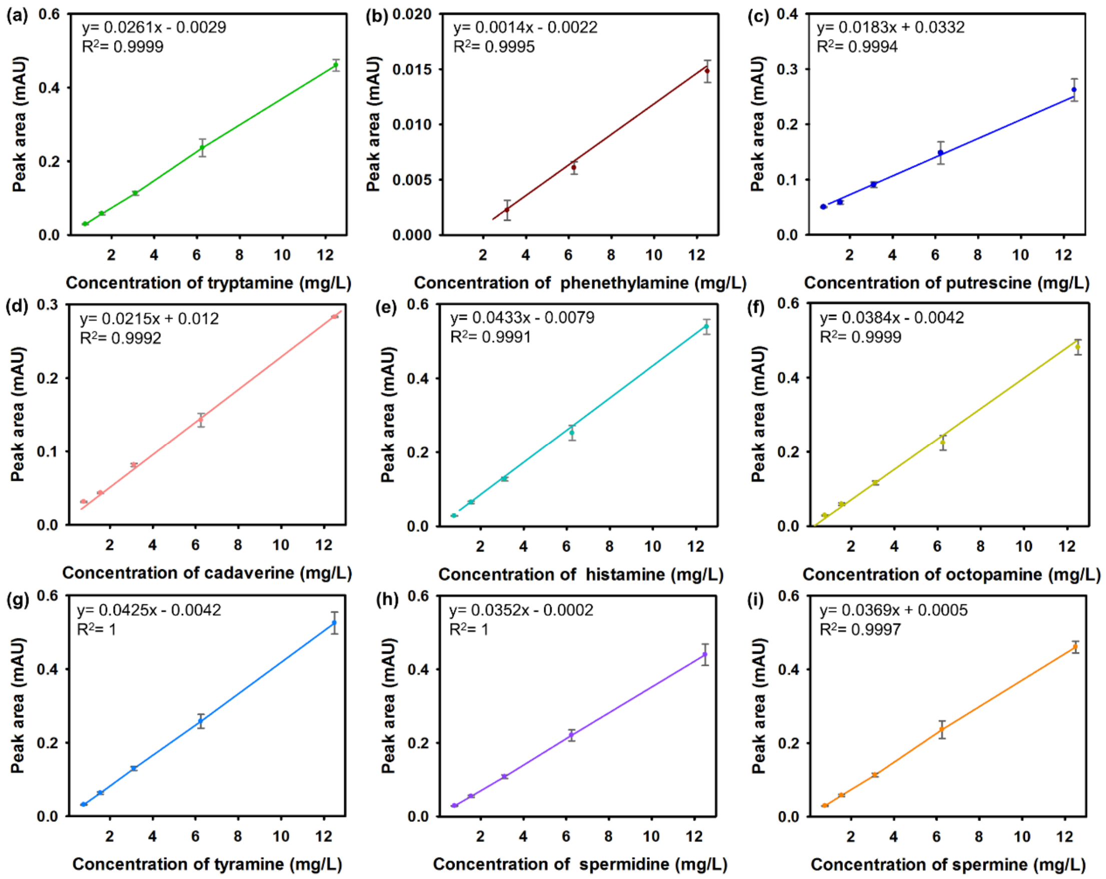

Figure S8. Standard curves of nine standard BAs with peak areas against the concentrations of standard BAs.

Table S1. Changes of BAs in chicken during storage and corresponding ΔE and FI_{570nm}/FI_{410nm} of H^{+}MQ-loaded paper chip.

Storage time 25°C (h)	Tyramine (mg/kg)	Putrescine (mg/kg)	Histamine (mg/kg)	Cadaverine (mg/kg)	BAI (mg/kg)	Total (mg/kg)	ΔE	FI_{570nm}/FI_{410nm}
0	ND	ND	ND	ND	ND	9.24±1.35	2.80±0.40	0.009±0.001
2	2.12±0.43	1.96±0.02	ND	ND	4.08±0.76	94.04±7.41	5.98±0.80	0.011±0.001
3	2.91±0.72	1.69±0.15	ND	ND	4.60±0.37	115.02±10.59	9.26±0.69	0.015±0.001
4	3.61±0.82	4.04±1.03	ND	ND	7.65±1.04	137.51±12.83	11.29±1.20	0.024±0.001
5	4.33±0.33	5.03±0.45	ND	ND	9.36±0.95	156.35±11.35	13.37±1.00	0.031±0.001
6	5.04±1.18	7.10±2.22	ND	ND	12.14±2.04	184.62±10.13	15.63±1.80	0.046±0.001
7	6.87±0.59	8.63±1.28	ND	ND	15.50±2.73	215.63±12.59	18.05±1.41	0.059±0.001
8	8.93±2.43	9.54±2.03	ND	ND	18.47±2.05	230.73±17.93	20.67±2.11	0.062±0.001
10	13.92±2.51	20.64±4.63	ND	ND	34.56±3.04	319.64±30.61	21.45±1.78	0.073±0.002
11	24.91±5.41	26.32±3.74	ND	ND	51.23±2.32	332.22±12.56	23.17±2.10	0.078±0.002
12	48.94±6.05	30.71±4.62	ND	ND	79.65±5.78	359.51±29.62	25.88±2.01	0.081±0.002
13	55.57±3.76	32.46±5.27	ND	ND	88.03±5.69	399.63±15.18	28.66±1.03	0.084±0.002
16	82.05±8.01	43.84±4.06	ND	ND	124.89±8.30	461.40±35.96	30.66±1.23	0.090±0.002
19	90.95±8.29	50.45±6.58	ND	ND	141.40±6.40	518.55±20.24	31.28±1.16	0.095±0.002
20	95.43±7.41	56.01±3.01	ND	ND	151.44±9.42	639.60±40.71	31.66±2.01	0.098±0.002
23	106.72±8.51	60.38±8.81	ND	ND	167.10±9.66	764.40±21.20	31.80±1.59	0.112±0.002
24	128.06±9.61	67.14±5.17	ND	ND	195.2±8.58	803.19±58.72	31.70±1.29	0.110±0.002

Note: The red highlight indicated the food spoilage with the BAI value over 50 mg/kg; "ND" indicated "not detected".

Table S2. Changes of BAs in weeverfish during storage and corresponding ΔE and FI_{570nm}/FI_{410nm} of $H^{+}MQ$-loaded paper chip.

Storage time 25°C (h)	Tyramine (mg/kg)	Putrescine (mg/kg)	Histamine (mg/kg)	Cadaverine (mg/kg)	BAI (mg/kg)	Total (mg/kg)	ΔE	FI_{570nm}/FI_{410nm}
0	ND	ND	ND	ND	ND	23.26±3.45	3.52±1.28	0.009±0.001
5	8.86±1.15	3.55±0.57	4.79±0.24	6.56±1.28	23.76±2.56	95.95±1.56	23.81±1.07	0.074±0.003
6	12.12±1.83	4.45±1.06	6.36±1.48	7.03±1.05	29.96±3.89	103.62±8.54	25.56±1.34	0.081±0.002
8	20.71±3.20	6.15±1.58	15.33±3.24	12.83±3.21	55.02±4.31	130.03±4.15	28.90±1.24	0.085±0.003
9	50.87±5.87	8.95±1.28	21.58±2.88	16.89±2.62	98.29±11.10	168.50±14.20	30.53±2.04	0.092±0.002
10	68.42±2.08	9.62±1.77	40.67±5.89	23.94±2.55	142.65±12.90	193.36±13.00	31.77±1.05	0.105±0.002
12	80.39±8.40	11.59±1.42	62.47±5.69	40.58±2.00	195.03±10.28	254.18±20.17	33.03±1.55	0.112±0.002
13	94.73±8.25	23.39±3.45	72.06±8.24	44.31±3.14	234.49±16.77	281.91±15.45	33.89±1.60	0.115±0.003
17	152.56±7.92	48.29±3.25	84.27±5.24	79.00±6.24	364.12±25.67	390.54±18.21	33.49±1.47	0.120±0.002
19	176.23±6.25	94.65±4.25	97.40±5.29	143.74±10.65	512.02±16.61	528.48±25.29	32.79±2.18	0.125±0.003

Note: The red highlight indicated the food spoilage with the BAI value over 50 mg/kg; "ND" indicated "not detected".

Table S3. Changes of BAs in shrimp during storage and corresponding ΔE and FI_{570nm}/FI_{410nm} of H^{+}MQ-loaded paper chip.

Storage time 25°C (h)	Tyramine (mg/kg)	Putrescine (mg/kg)	Histamine (mg/kg)	Cadaverine (mg/kg)	BAI (mg/kg)	Total (mg/kg)	ΔE	FI_{570nm}/FI_{410nm}
0	ND	ND	ND	ND	ND	1.24±0.21	4.39±0.87	0.009±0.001
3	3.62±0.45	2.90±0.45	2.30±0.54	3.20±0.21	12.02±1.67	43.87±6.20	21.14±2.90	0.072±0.003
4	4.57±0.87	5.84±0.58	3.02±0.53	7.89±1.08	21.32±4.98	55.27±3.49	22.25±1.41	0.073±0.002
5	5.34±1.12	6.60±0.56	5.40±0.91	13.60±1.03	30.95±3.23	65.34±5.45	23.12±1.22	0.074±0.003
6	6.58±1.13	12.78±2.85	6.01±0.70	20.80±2.61	46.26±4.13	75.43±8.30	24.43±1.06	0.078±0.002
7	7.55±1.63	22.24±3.47	8.60±1.47	28.89±1.45	67.28±5.48	82.48±7.96	25.36±0.91	0.081±0.004
8	8.37±1.62	35.15±7.01	13.90±2.03	55.81±8.36	113.23±8.03	146.21±8.51	28.51±1.21	0.091±0.002
10	9.42±0.68	73.67±9.14	23.66±4.27	195.67±10.14	302.42±23.56	322.33±22.48	31.51±0.91	0.112±0.003
12	14.64±3.80	140.49±10.42	58.47±8.19	240.71±17.02	454.31±36.09	575.61±41.93	31.47±0.88	0.111±0.003
13	15.11±2.56	155.33±16.54	81.69±10.28	302.10±20.14	554.23±29.38	629.96±17.14	31.29±1.46	0.110±0.004
14	19.13±2.45	216.69±20.21	109.59±15.14	355.21±18.14	700.62±52.99	869.66±78.15	31.54±1.29	0.112±0.005

Note: The red highlight indicated the food spoilage with the BAI value over 50 mg/kg; "ND" indicated "not detected".

Table S4. Changes of BAs in chicken during storage and corresponding ΔE and FI_{570nm}/FI_{410nm} of $H^{+}MQ$-loaded paper chip.

Storage time 4°C (d)	Tyramine (mg/kg)	Putrescine (mg/kg)	Histamine (mg/kg)	Cadaverine (mg/kg)	BAI (mg/kg)	Total (mg/kg)	ΔE	FI_{570nm}/FI_{410nm}
0	ND	ND	ND	ND	ND	9.24±0.35	4.57±0.67	0.009±0.001
1	3.04±0.22	3.87±0.68	2.87±0.17	1.99±0.11	11.77±2.57	50.87±4.80	20.39±1.05	0.064±0.002
2	3.01±0.69	7.82±1.57	3.54±0.39	2.07±0.27	16.44±4.73	77.26±1.41	23.18±1.28	0.071±0.002
3	4.58±0.59	13.87±1.53	4.87±0.88	3.47±0.18	26.79±6.02	89.27±6.87	25.08±1.34	0.079±0.002
4	5.01±0.95	15.69±3.58	7.60±1.87	5.10±0.83	33.40±1.81	120.20±0.85	32.25±1.32	0.112±0.003
5	8.54±0.94	21.74±3.45	15.10±2.84	9.87±1.29	55.25±9.02	114.09±8.41	32.65±0.64	0.114±0.003
6	12.41±2.39	66.21±10.55	63.41±5.36	17.81±6.64	159.84±6.24	334.75±7.25	33.25±1.20	0.110±0.004
7	15.89±3.10	90.57±6.08	138.06±9.57	45.89±6.78	290.41±16.28	450.85±27.00	33.74±1.67	0.112±0.002
8	38.51±6.25	166.26±20.41	182.35±12.52	114.12±32.59	501.24±17.94	1223.42±18.17	34.41±1.37	0.114±0.005
9	48.10±6.17	220.84±12.08	208.97±10.08	169.40±9.07	647.31±34.26	1589.10±92.01	34.07±1.53	0.114±0.003
10	67.82±10.84	414.36±30.58	384.26±20.38	422.32±32.43	1288.76±23.56	2362.85±105.58	34.14±1.28	0.116±0.005

Note: The red highlight indicated the food spoilage with the BAI value over 50 mg/kg; "ND" indicated "not detected".

Table S5. Changes of BAs in weeverfish during storage and corresponding ΔE and FI_{570nm}/FI_{410nm} of $H^{+}MQ$-loaded paper chip.

Storage time 4°C (d)	Tyramine (mg/kg)	Putrescine (mg/kg)	Histamine (mg/kg)	Cadaverine (mg/kg)	BAI (mg/kg)	Total (mg/kg)	ΔE	FI_{570nm}/FI_{410nm}
0	ND	ND	ND	ND	ND	23.26±3.45	3.80±1.60	0.009±0.001
1	2.16±0.38	2.87±0.51	ND	ND	5.03±1.00	36.81±2.89	18.72±0.77	0.062±0.002
2	4.53±0.69	15.69±1.58	3.87±0.59	1.56±0.19	25.65±0.76	58.86±8.52	24.69±2.12	0.070±0.002
3	10.59±2.78	30.87±6.84	8.52±1.57	6.90±1.08	56.88±6.58	182.07±10.18	29.63±2.15	0.091±0.002
4	58.58±7.59	87.51±9.20	20.37±3.16	39.14±4.81	205.60±10.19	265.04±10.08	32.19±2.18	0.112±0.002
5	79.84±5.39	128.59±8.16	40.58±3.89	68.21±7.84	257.22±18.07	348.01±28.59	33.64±1.99	0.122±0.002
6	103.81±12.37	151.27±12.53	65.30±9.42	133.10±10.58	453.48±11.23	542.43±15.45	34.18±1.20	0.114±0.003
8	343.52±36.16	336.58±30.28	205.69±11.28	308.14±28.14	1193.93±66.47	1555.88±16.59	33.59±2.94	0.113±0.006
10	568.54±35.34	861.84±33.47	361.08±34.40	734.02±33.48	2525.48±94.17	3351.29±86.48	33.94±2.17	0.119±0.005

Note: The red highlight indicated the food spoilage with the BAI value over 50 mg/kg; "ND" indicated "not detected".

Table S6. Changes of BAs in shrimp during storage and corresponding ΔE and FI_{570nm}/FI_{410nm} of H^{+}MQ-loaded paper chip.

Storage time 4°C (d)	Tyramine (mg/kg)	Putrescine (mg/kg)	Histamine (mg/kg)	Cadaverine (mg/kg)	BAI (mg/kg)	Total (mg/kg)	ΔE	FI_{570nm}/FI_{410nm}
0	ND	ND	ND	ND	ND	ND.	4.57±0.67	0.009±0.001
1	2.96±0.47	4.36±0.58	4.03±0.63	ND	11.35±1.89	98.25±7.05	16.83±0.73	0.052±0.002
2	5.18±0.69	12.67±1.57	12.27±1.39	15.47±2.83	45.60±4.62	160.50±12.39	26.26±3.50	0.072±0.003
3	24.35±3.80	18.45±3.16	19.56±2.08	27.65±5.49	90.01±18.07	193.88±15.75	30.66±2.13	0.106±0.004
4	35.89±2.95	56.73±3.58	34.93±1.87	72.98±6.27	200.53±13.67	282.48±23.28	34.47±1.13	0.122±0.006
5	45.87±4.67	90.48±2.49	130.80±8.06	89.28±6.41	356.43±26.17	382.04±31.07	34.50±2.25	0.125±0.004
6	73.18±5.39	114.14±10.55	159.25±5.36	172.54±10.64	519.11±17.00	622.33±36.21	35.15±1.20	0.115±0.004
7	95.01±7.14	254.18±12.18	200.18±15.91	219.20±18.64	768.57±51.06	891.23±66.17	34.64±2.04	0.116±0.004
8	167.41±10.25	299.10±20.41	268.07±22.52	312.04±32.59	1046.62±36.44	1529.96±75.37	34.90±1.57	0.116±0.003
9	183.18±10.95	480.29±22.80	493.28±38.49	150.69±7.52	1307.44±86.19	1954.25±98.34	35.17±1.44	0.125±0.004
10	255.18±15.84	542.80±30.58	529.21±20.38	493.52±32.43	1672.79±61.81	2269.66±120.19	34.58±2.97	0.129±0.008

Note: The red highlight indicated the food spoilage with the BAI value over 50 mg/kg; "ND" indicated "not detected".

Table S7. Changes of BAs in chicken during storage and corresponding ΔE and FI_{570nm}/FI_{410nm} of H^+MQ-loaded paper chip.

Storage time -20°C (d)	Tyramine (mg/kg)	Putrescine (mg/kg)	Histamine (mg/kg)	Cadaverine (mg/kg)	BAI (mg/kg)	Total (mg/kg)	ΔE	FI_{570nm}/FI_{410nm}
0	ND	ND	ND	ND	ND	9.24±2.35	3.19±1.01	0.008±0.001
7	3.81±0.27	2.85±0.41	2.98±0.29	ND	9.64±1.32	28.84±4.10	3.70±1.29	0.008±0.003
14	4.68±0.57	0.79±0.13	2.84±0.66	ND	8.31±1.21	26.02±3.31	5.43±1.23	0.009±0.001
21	4.63±0.99	0.53±0.09	2.52±0.87	ND	7.68±1.10	28.31±2.14	7.84±2.77	0.010±0.002
28	5.04±1.61	0.70±0.11	2.68±0.72	ND	8.42±1.63	28.59±3.71	6.64±1.51	0.012±0.001
35	8.17±1.42	0.56±0.04	2.56±0.91	ND	11.29±2.48	27.20±3.10	7.92±1.37	0.013±0.001

Note: The red highlight indicated the food spoilage with the BAI value over 50 mg/kg; "ND" indicated "not detected".

Table S8. Changes of BAs in weeverfish during storage and corresponding ΔE and FI_{570nm}/FI_{410nm} of H^+MQ-loaded paper chip.

Storage time -20°C (d)	Tyramine (mg/kg)	Putrescine (mg/kg)	Histamine (mg/kg)	Cadaverine (mg/kg)	BAI (mg/kg)	Total (mg/kg)	ΔE	FI_{570nm}/FI_{410nm}
0	ND	ND	ND	ND	ND	23.26±3.45	3.69±0.92	0.009±0.001
7	11.21±0.24	ND	1.54±0.27	ND	12.75±0.26	74.15±1.59	2.74±1.62	0.008±0.001
14	12.94±0.91	ND	5.87±0.67	ND	18.81±0.79	73.11±1.54	8.53±2.06	0.010±0.001
21	22.67±2.17	ND	6.58±2.60	ND	29.25±2.39	90.22±2.17	7.17±1.26	0.011±0.002
28	16.68±2.64	ND	10.69±1.52	ND	27.37±2.08	117.97±2.99	9.77±1.91	0.012±0.001
35	25.31±3.97	ND	18.54±2.87	ND	43.85±3.42	157.94±4.27	9.77±1.04	0.011±0.002

Note: The red highlight indicated the food spoilage with the BAI value over 50 mg/kg; "ND" indicated "not detected".

Table S9. Changes of BAs in shrimp during storage and corresponding ΔE and FI_{570nm}/FI_{410nm} of H^+MQ-loaded paper chip.

Storage time at -20 °C (d)	Tyramine (mg/kg)	Putrescine (mg/kg)	Histamine (mg/kg)	Cadaverine (mg/kg)	BAI (mg/kg)	Total (mg/kg)	ΔE	FI_{570nm}/FI_{410nm}
0	ND	ND	ND	ND	0	1.24±0.01	2.94±0.50	0.008±0.001
7	3.50±0.58	ND	3.78±0.95	ND	7.28±0.77	105.17±5.39	3.52±1.32	0.008±0.001
14	6.70±1.25	ND	4.84±0.81	ND	11.54±1.03	92.76±7.28	8.81±1.08	0.011±0.001
21	8.14±1.89	ND	6.72±1.49	ND	14.86±1.69	131.86±13.59	9.25±1.66	0.012±0.001
28	14.35±3.57	ND	5.98±1.27	ND	20.33±2.42	130.75±9.48	9.47±1.83	0.012±0.001
35	18.64±3.67	ND	6.90±0.58	ND	25.54±3.96	152.72±14.58	10.10±2.02	0.013±0.002

Note: The red highlight indicated the food spoilage with the BAI value over 50 mg/kg; "ND" indicated "not detected".

Table S10. Comparison of the proposed sensing system with other reported methods for BAs detection.

Underlying mechanism	Target	Sensor type	Signal	Qualitative/ Quantitative	Ref.
BAs induce AuNPs aggregation.	Histamine	Solution-based	Colorimetric	Quantitative	[1]
MAF exhibits high selectivity for primaryand secondary amines.	Amine vapors	Solution-based and solid-based	Colorimetric	Quantitative	[2]
Amine vapors trigger β-D-glucose to reduce Ag^{+} to AgNPs.	Amine vapors	Hydrogels	Colorimetric	Both	[3]
Amine vapors react with BZCO to generate the coumarin fluorophore.	Amine vapors	Paper-based	Fluorescent	Both	[4]
An intermolecular charge transfer complex was formed between the polymer and analyte.	Ethylenediamine, putrescine and cadaverine	Polymer film-based	Colorimetric	Quantitative	[5]
H^{+}DQ2 can be deprotonated to form yellow fluorescent DQ2 by biogenic amine.	Amine vapors	Paper-based	Fluorescent	Semi-quantitative	[6]
Anthocyanins are sensitive to volatile ammonia and pH.	Amine vapors	Film-based	Colorimetric	Qualitative	[7]
H^{+}MQ can be deprotonated to form fluorescent MQ by biogenic amine.	Amine vapor	Paper-based	Colorimetric /Fluorescent	Both	this work
